# Visceral Adiposity Index May Be a Surrogate Marker for the Assessment of the Effects of Obesity on Arterial Stiffness

**DOI:** 10.1371/journal.pone.0104365

**Published:** 2014-08-08

**Authors:** Fan Yang, Guofeng Wang, Zhixiao Wang, Min Sun, Mengdie Cao, Zhenxin Zhu, Qi Fu, Jia Mao, Yun Shi, Tao Yang

**Affiliations:** Department of Endocrinology, First Affiliated Hospital of Nanjing Medical University, Nanjing, Jiangsu, China; University of Bari Aldo Moro, Italy

## Abstract

**Objective:**

The relationship between obesity and cardiovascular disease (CVD) remains unclear. This study aims to describe the relationship between arterial stiffness and obesity in order to investigate the effects of obesity on CVD.

**Methods:**

We collected data from 5,158 individuals over 40 years of age from a cross-sectional study in Nanjing, China. Anthropometric, demographic, hemodynamic measurements and arterial stiffness measured through brachial-ankle pulse wave velocity (baPWV) were obtained. Subjects were grouped by body mass index (BMI), waist circumference (WC) and visceral adiposity index (VAI), a sex-specific index based on BMI, WC, triglyceride (TG) and high-density lipoprotein cholesterol (HDL-C).

**Results:**

The multivariate regression analysis revealed a negative but weak effect of BMI (β = −0.047, P<0.001) on baPWV, but failed to demonstrate any significant effect of WC on baPWV while VAI was a positive independent indicator of baPWV (β = 0.023, P = 0.022). The unadjusted baPWV significantly increased across groups with higher obesity categories (P<0.01). Although the positive association was lost after adjustments for confounding factors in the BMI or WC categories (P>0.05), it was still obtained between baPWV and VAI quartile (P<0.01). No differences were observed among the metabolically healthy groups or the metabolically abnormal groups in the BMI and WC categories (P>0.05). However, baPWV significantly increased across groups with higher VAI categories even in the same metabolic category (P<0.01).

**Conclusions:**

This study supports the concept of heterogeneity of metabolic status among individuals within the same obesity range. Obese individuals are at an increased risk of arterial stiffness regardless of their metabolic conditions. VAI may be a surrogate marker for the assessment of obesity and the effects of obesity on arterial stiffness.

## Introduction

The prevalence of obesity is increasing at a dramatic rate in developed and developing countries, with data indicating that this will affect more than one billion people by 2030 [1]. Many studies have firmly recognized obesity as an independent determinant of cardiovascular diseases (CVD) [2]–[5]. Body mass index (BMI) and waist circumference (WC) are commonly recommended for the assessment of obesity [6], [7]. However, recent studies have challenged the relationship between obesity assessed by BMI or WC and CVD [8], [9]. Furthermore, the metabolically healthy but obese (MHO) phenotype, characterized by obesity with a favorable metabolic profile [10], has been proposed and was reported to hold a high prevalence among obese individuals [11]. Nevertheless, the relationship between obesity and CVD has not been established [12]–[14].

Recent studies have shown that visceral adipose tissue participates in the pathogenesis of CVD [15], [16]. Visceral adiposity index (VAI), based on WC, BMI, triglyceride (TG) and high-density lipoprotein cholesterol (HDL-C), is a newly proposed sex-specific indicator of fat distribution and function associated with cardiometabolism[17]. Arterial stiffness, indirectly measured through non-invasive pulse wave velocity (PWV), is one of the pathological states of vascular damage, contributing to both cardiovascular morbidity and mortality [18]. Although aortic PWV is accurate and reproducible, it may not be suitable for routine clinical practice because the use of pressure transducers or Doppler probes on target arteries may be perceived as somewhat difficult for clinical staff. In addition, some subjects may feel uncomfortable exposing the inguinal area. Therefore, brachial-ankle pulse wave velocity (baPWV) is considered an ideal technique to evaluate arterial stiffness, albeit not the gold standard. In this study, we describe the relationship between arterial stiffness (assessed by baPWV) and obesity (assessed by BMI, WC and VAI) to help to explore the effects of obesity on CVD.

## Methods

### Study population

A total of 5,158 community-dwelling individuals over 40 years of age living in the Gulou district, Nanjing, Jiangsu Province, China, from June 2011 to December 2011, were recruited for the study. The study was approved by the institutional review board of the First Affiliated Hospital of Nanjing Medical University and all participants provided written informed consent before initiation of the study protocol.

### Anthropometric and demographic measurements

A questionnaire was used to obtain information about demographic factors, medical history, medication use, and personal health habits, such as smoking habits. Anthropometric parameters were obtained by trained technicians using standard methods. Standing height was measured to the nearest millimeter, without shoes, using a wall-mounted vertical ruler. Weight was assessed to the nearest 0.1 kg without outdoor clothing or shoes. BMI was calculated as the weight in kilograms divided by the square of the height in meters. WC was measured in the standing position as the midpoint between the lower rib margin and the iliac crest according to the WHO's recommendation [19]. The cut-off points for BMI and WC were proposed by the Working Group on Obesity in China (WGOC) based on large sample epidemiological surveys [20].According to BMI, individuals were classified as overweight (BMI≥24 and <28 kg/m^2^), or obese (BMI≥28 kg/m^2^). According to WC, individuals were divided into three groups with the cut-off points of 85 cm(male)/80 cm(female) and 90 cm(male)/85 cm(female).

### Laboratory measurements

Venous blood samples were taken after an overnight fast for measurement of fasting plasma glucose (FPG), HbA1c, TG, cholesterol (CHOL), HDL-C and low-density lipoprotein cholesterol (LDL-C). A 75 g oral glucose tolerance test was performed and blood samples were taken at 0, 30, and 120 min for the measurement of plasma glucose and insulin concentrations. All measurements were analyzed by an autoanalyzer (Modular E170; Roche). Serum insulin concentrations were determined with the Phadebas Insulin Test (Pharmacia, Uppsala, Sweden) using a radioimmunosorbent technique. Insulin resistance was estimated using homeostasis model assessment-insulin resistance (HOMA-IR) calculated as fasting plasma insulin (µU/ml) × fasting plasma glucose (mmol/L)/22.5 [21]. The Matsuda Insulin Sensitivity Index (Matsuda ISI), an index of whole-body insulin sensitivity was calculated using the following formula: 10,000/[(G0×I0)×(G^−^×I^−^)]∧0.5 [22]. Diabetes is defined as a fasting glucose≥7.0 mmol/l or 2 h glucose≥11.1 mmol/L. The metabolic healthy phenotype is defined as the simultaneous presence of a HDL-C level of at least 1.04 mmol/l and the absence of type 2 diabetes and hypertension [23]. VAI, a sex-specific index based on WC, BMI, TG and HDL-C, was calculated as follows [17]:

Males: VAI = (WC/(39.68+(1.88×BMI)))×(TG/1.03)×(1.31/HDL)

Females: VAI = (WC/(36.58+(1.89×BMI)))×(TG/0.81)×(1.52/HDL)

### Blood pressure and baPWV

Blood pressure (BP) was measured in a seated position using a standard mercury sphygmomanometer after a 5–10-min rest period under quiet environmental conditions according to a standardized protocol. BP measurement was repeated three times at 5 min intervals by a trained physician. Average BP was then calculated from the three consecutive measurements. Hypertension is defined as an average systolic BP (SBP) ≥140 mmHg or diastolic BP (DBP) ≥90 mmHg. BaPWV was measured in the supine position after at least 5 min of bed rest using a volume-plethysmographic apparatus with an automatic waveform analyzer (VP-1000; Colin Co., Komaki, Japan), which simultaneously records PWV, the electrocardiogram and heart sounds, and was calculated as distance/time (cm/s). The distance between sampling points of the baPWV was calculated automatically according to the height of the subjects. The time interval between the brachium and the ankle was defined as the time interval between the wave front of the brachial waveform and that of the ankle waveform. Mean values of right and left baPWV were used for analysis.

### Statistical analysis

Continuous data were expressed as mean ± s.d., while categorical data were described as frequencies or percentages. The analyses of continuous variables to assess the differences among obesity categories were determined using ANOVA, followed by Bonferroni's test for multiple comparisons. Step-wise multiple linear regression analysis was performed to determine the correlation and independent variables for baPWV. Variables selected for multivariate regression were based on their clinical and biological plausibility and after a significant correlation had been obtained. PG120 was found to be a stronger independent predictor of arterial stiffness than FPG [24], [25]. We analyzed the correlation between age, smoking, SBP, DBP, HR, BMI, WC, VAI, PG120, HDL-C, LDL-C, TG, CHOL and baPWV. Smoking was not significantly correlated with baPWV. Therefore, the remaining variables entered the multiple regression model. Univariate general linear model was used to adjust for confounding factors. A two-tailed P<0.05 was considered to be statistically significant. The statistical analyses were conducted using the SPSS 17.0 for Windows (Chicago, IL, USA).

## Results

The clinical and laboratory characteristics of 5,158 subjects are presented in Table 1. Overall, 12.3% and 39.7% of the participants had a BMI of >27.9 kg/m^2^ and >23.9 kg/m^2^, respectively. The prevalence of individuals with a WC >85 cm(male)/80 cm(female) and >90 cm(male)/85 cm(female) was 23.4% and 39.4%, respectively.

**Table 1:** Clinical and laboratory characteristics.

Variables	
**Age(years)**	59.9±9.1
**Female(%)**	57.8
**Smoker(%)**	24.9
**BMI(kg/m^2^)**	24.3±3.2
**WC(cm)**	84.3±9.5
**SBP(mmHg)**	130±18
**DBP(mmHg)**	79±11
**HR(bpm)**	78±11
**FPG(mmol/l)**	6.1±1.6
**PG30(mmol/l)**	9.7±2.3
**PG120(mmol/l)**	8.5±3.7
**INS0(µU/ml)**	12.9±12.5
**INS30(µU/ml)**	62.6±54.9
**INS120(µU/ml)**	64.8±58.6
**HDL-C(mmol/l)**	1.3±0.3
**LDL-C(mmol/l)**	2.9±0.8
**CHOL(mmol/l)**	4.9±1.0
**TG(mmol/l)**	1.6±1.1
**VAI**	2.2±2.2
**baPWV(cm/s)**	1569.8±350.9
**Hypertension(%)**	37.8
**Diabetes(%)**	26.4

Variables are expressed as frequency in percent or means ± s.d.

BMI, body mass index; WC, waist circumference; SBP, systolic blood pressure; DBP, diastolic blood pressure; HR, heart rate; FPG, fasting plasma glucose; PG30, 30-minute post-OGTT plasma glucose; PG2h, 120-minute post-OGTT plasma glucose; INS0, fasting serum insulin; INS30, 30-minute post-OGTT serum insulin; INS120, 120-minute post-OGTT serum insulin; HDL-C, high-density lipoprotein cholesterol; LDL-C, low-density lipoprotein cholesterol; CHOL, cholesterol; TG, triglyceride; baPWV, brachial-ankle pulse wave velocity.

The step-wise multivariate regression analysis was performed to assess the correlation of baPWV with other clinical and laboratory variables in all subjects. Table 2 shows that, for model 1 with BMI, SBP, age, heart rate (HR), 2 hours post-OGTT plasma glucose (PG120), TG and LDL-C were positive independent predictors of baPWV (P<0.05), HDL-C was negatively associated with baPWV (P = 0.002). It was noteworthy that BMI was also a negative but weak predictor of baPWV (β = −0.047, P<0.001). Moreover, a multivariate regression analysis using WC instead of BMI (Table 2, model 2) failed to demonstrate any significant effect of the parameter on baPWV. However, the model using VAI instead of BMI or WC (Table 2, model 3) revealed a positive independent effect of the parameter on baPWV (β = 0.023, P = 0.022). SBP explained 35% of the baPWV variability.

**Table 2:** Results of step-wise multiple regression analysis to assess the correlation of brachial-ankle pulse wave velocity with other variables in all subjects.

Variables	*β*	*P* value	R^2^	R^2^ change
**Model 1 (With BMI)**				
**SBP**	0.438	<0.001	0.350	0.350
**Age**	0.374	<0.001	0.479	0.130
**HR**	0.162	<0.001	0.509	0.030
**PG120**	0.095	<0.001	0.518	0.009
**BMI**	−0.047	<0.001	0.519	0.001
**TG**	0.026	0.0012	0.520	0.001
**HDL-C**	−0.035	0.002	0.521	0.001
**LDL-C**	0.023	0.025	0.521	0.000
**Model 2 (With WC)**				
**SBP**	0.428	<0.001	0.350	0.350
**Age**	0.378	<0.001	0.479	0.129
**HR**	0.163	<0.001	0.509	0.030
**PG120**	0.095	<0.001	0.518	0.009
**TG**	0.028	0.005	0.519	0.001
**Model 3 (With VAI)**				
**SBP**	0.429	<0.001	0.349	0.350
**Age**	0.377	<0.001	0.479	0.129
**HR**	0.164	<0.001	0.509	0.030
**PG120**	0.096	<0.001	0.518	0.009
**VAI**	0.023	0.022	0.518	0.000

SBP, systolic blood pressure; HR, heart rate; PG120, 120-minute post-OGTT plasma glucose; BMI, body mass index; TG, triglyceride; HDL-C, high-density lipoprotein cholesterol; LDL-C, low-density lipoprotein cholesterol; WC, waist circumference; VAI, visceral adiposity index.

**Figure 1:**
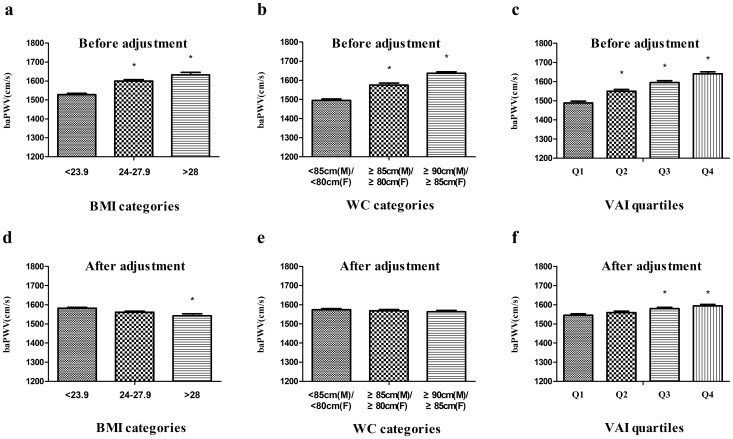
Relationship between obesity category (based on BMI, waist circumference and visceral adiposity index) and brachial-ankle pulse wave velocity (mean ± s.e.). a, b, c: Before adjustments. d, e: After adjustments for systolic blood pressure (SBP), age, heart rate, 120-min post-OGTT plasma glucose (PG120) and lipids. f: After adjustments for SBP, age, heart rate, PG120, low-density lipoprotein cholesterol and cholesterol. * P<0.01 for the comparison to the first category or first visceral adiposity index quartile.

The relationship between obesity category (based on BMI, WC and VAI) and baPWV is showed in Figure 1. The unadjusted baPWV significantly increased across groups with higher obesity categories (Fig 1a, b, c; P<0.01). After adjusting for SBP, age, HR, PG120 and lipids, the positive association disappeared in the BMI and WC categories (Fig 1d, e; P>0.05) but was still observed between baPWV and VAI quartile (Fig 1f; P<0.01).

**Figure 2:**
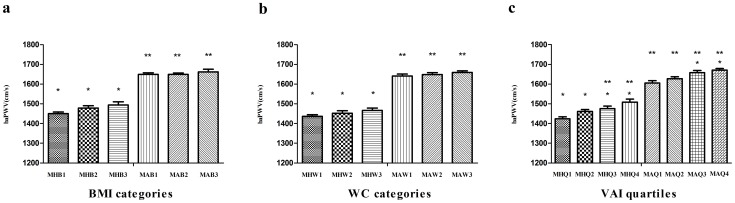
Association between body size phenotypes (combined consideration of BMI or waist circumference categories or visceral adiposity index quartiles and the absence or presence of the abnormal metabolic components) and brachial-ankle pulse wave velocity (mean ± s.e.) after adjustments for age and heart rate. MH, metabolically healthy individuals; MA, metabolically abnormal individuals; baPWV, brachial-ankle pulse wave velocity. * P<0.01 for the comparison to the metabolically abnormal first category; ** P<0.01 for the comparison to the metabolically healthy first category.

In order to further assess the effect of obesity on arterial stiffness, body size phenotypes were defined based on the combined consideration of the absence or presence of the abnormal metabolic components and the obesity categories. Compared with the metabolically abnormal category, metabolically healthy individuals had a lower value of baPWV regardless of their obesity category (Fig 2a, b, c; P<0.01). Similarly, the metabolically abnormal individuals had an unfavorable value in baPWV compared to the metabolically healthy category (Fig 2a, b, c; P<0.01). No differences were observed among the metabolically healthy groups or the metabolically abnormal groups in the BMI and WC category (Fig 2a, b; P>0.05). However, baPWV significantly increased across groups with higher VAI categories in the same metabolic category (Fig 2c; P<0.01).

**Figure 3:**
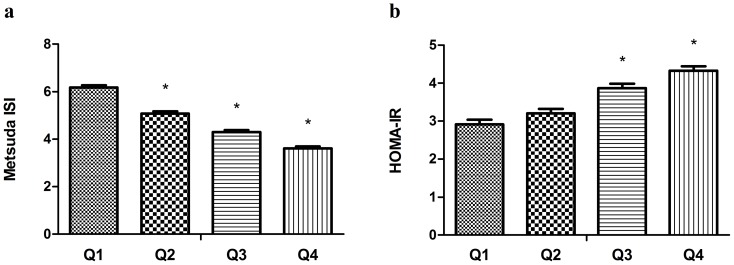
Matsuda ISI (mean ± s.e.) and HOMA-IR (mean ± s.e.) according to visceral adiposity index quartiles after adjustments for sex and age. * P<0.001 for the comparison to the first quartile. Matsuda ISI, Matsuda Insulin Sensitivity Index; HOMA-IR, homeostasis model assessment of insulin resistance.

The associations between HOMA-IR, Matsuda ISI and VAI category are depicted in Figure 3. Individuals with a higher VAI category had lower insulin sensitivity (Fig 3a; P<0.001) and greater insulin resistance (Fig 3b; P<0.001). Furthermore, SBP and DBP were significantly elevated with increasing VAI category (P<0.001) (data not shown).

## Discussion

Our study presents new data investigating the relationship between obesity and arterial stiffness. BMI and WC are not independent predictors of arterial stiffness. VAI, an indirect indicator of fat distribution and function, is independently associated with increased arterial stiffness, even in metabolically healthy individuals, and may be suitable as an evaluation index of the effects of obesity on arterial stiffness.

A population-based study has demonstrated that BMI and WC are not related to aortic stiffness in the fully adjusted model [26]. More recent data from a Brazilian young and middle-aged population (25–64 years of age) indicated that BMI and WC are not independently related to increased aortic stiffness [13]. Similarly, the results were also confirmed in our study of middle-aged and elderly population indicating that BMI and WC were not independent predictors of arterial stiffness. Thus, these results raise doubts of the comprehensiveness of classical obesity measurements for the prediction of the effects of obesity on arterial stiffness.

Both BMI and WC are clinical parameters reflecting the total visceral and subcutaneous fat mass [7], and so cannot reflect only subcutaneous or only visceral fat mass [27], the latter being the key factor in metabolic alteration development [28]–[32]. Considering that the use of WC as a surrogate for visceral fat is controversial and that MRI is costly and inconvenient despite it being a well-established method to estimate visceral obesity, we have herein introduced the VAI. This index includes both anthropometric (BMI and WC) and metabolic (TG and HDL-C) parameters and is thought to be capable of indicating both fat distribution and function, which are not signified by BMI and WC separately [17]. VAI was significantly correlated to almost all metabolic syndrome factors and cardiovascular events; this trend was particularly apparent from the third VAI quartile. Our study confirmed the reported association between VAI and insulin resistance, and showed that baPWV significantly increased from the third VAI quartile even in the same metabolic category, indicating that VAI may be a surrogate marker evaluating the effects of obesity on arterial stiffness.

Although obesity has been widely recognized as an important risk factor for the development of CVD, to what extent the impact of obesity on arterial stiffness remains unknown. Arnlöv et al. [33] have shown that overweight and obese men without metabolic syndrome (MetS) have an increased risk of CVD during a 30-year follow-up. Conversely, an observational study [34] showed that the MHO individuals were not at an increased risk of CVD or all-cause mortality after 7 years of follow-up. Interestingly, our data seems to suggest that the presence of metabolic alterations, rather than obesity per se, is related to arterial stiffness.

A major confusion may have arisen from the difference in the follow-up duration. In the study by Arnlöv et al. [33], the Kaplan-Meier curves for healthy overweight and obese men without MetS began to diverge from the curve of healthy normal weight without MetS following a 10-year lag time. Two other studies [35], [36] with healthy obese individuals showed an impairment in endothelial function and early atherosclerosis assessed by flow-mediated dilation of the brachial artery and intima-media thickness of the common carotid artery (CCA-IMT). These suggest that metabolically healthy overweight and obese individuals already have some form of subtle target organ damage, which may require several decades to advance to CVD. Moreover, obesity has been shown to be a major factor responsible for the prevalence of metabolic alterations [37], metabolically healthy obesity has also been demonstrated to be a transient state [38], suggesting that obesity and metabolic abnormalities could not be viewed in isolation. These may also partially explain the small contribution of VAI to the variance of baPWV after adjusting for confounding factors.

We noted several strengths and limitations of our study. The advantages of this study include the larger population sample and it addresses not only findings related to total fat, but also to visceral fat and fat distribution indirectly reflected by VAI, a newly extrapolated index that may be used as a surrogate marker of adipose tissue dysfunction. However, our study is limited. First, the cross-sectional design of this study cannot directly establish any causal associations between obesity categories and arterial stiffness. Second, some (albeit small) portions of baPWV may be determined by ‘peripheral’ (or muscular) arterial stiffness, and baPWV is not the gold standard for the assessment of arterial stiffness. Nevertheless, previous studies have shown that there is a significant positive correlation between baPWV and CVD [39], [40]. Additionally, its technical simplicity, validity and reproducibility make it feasible for large population screening. Third, since the current study population had a high ratio of metabolic abnormality, the results might not be generalizable to all populations. However, it is still applicable to many populations worldwide since the incidence of metabolic abnormality is rapidly rising. Fourth, the only existing version of the VAI was modeled by Amato et al. [17] on a Caucasian population; thus, its suitability for other populations needs to be further investigated.

In conclusion, our study supports the concept of heterogeneity of metabolic status among individuals within the same obesity range. Obese individuals are at an increased risk of arterial stiffness regardless of their metabolic condition. Further studies are required in order to obtain a more suitable indicator to evaluate the impact of obesity on arterial stiffness. However, to a certain extent and considering the simplicity of the measurement and detection of the related indicators, VAI may be a surrogate marker for the assessment of obesity and the effects of obesity on arterial stiffness in daily clinical practice and population studies.
